# Fucoxanthin from Algae to Human, an Extraordinary Bioresource: Insights and Advances in up and Downstream Processes

**DOI:** 10.3390/md20040222

**Published:** 2022-03-23

**Authors:** Anne Pajot, Gia Hao Huynh, Laurent Picot, Luc Marchal, Elodie Nicolau

**Affiliations:** 1 Ifremer, GENALG Laboratory, Unité PHYTOX, F-44000 Nantes, France; giahaohuynh97@gmail.com (G.H.H.); elodie.nicolau@ifremer.fr (E.N.); 2 Unité Mixte de Recherche CNRS 7266 Littoral Environnement et Sociétés (LIENSs), Université La Rochelle, F-17042 La Rochelle, France; laurent.picot@univ-lr.fr; 3 Génie des Procédés Environnement (GEPEA), Université Nantes, F-44000 Saint Nazaire, France; luc.marchal@univ-nantes.fr

**Keywords:** fucoxanthin, golden-brown algae, *Tisochrysis lutea*, biosynthesis, downstream processes, extraction, centrifugal partition chromatography, global market

## Abstract

Fucoxanthin is a brown-colored pigment from algae, with great potential as a bioactive molecule due to its numerous properties. This review aims to present current knowledge on this high added-value pigment. An accurate analysis of the biological function of fucoxanthin explains its wide photon absorption capacities in golden-brown algae. The specific chemical structure of this pigment also leads to many functional activities in human health. They are outlined in this work and are supported by the latest studies in the literature. The scientific and industrial interest in fucoxanthin is correlated with great improvements in the development of algae cultures and downstream processes. The best fucoxanthin producing algae and their associated culture parameters are described. The light intensity is a major influencing factor, as it has to enable both a high biomass growth and a high fucoxanthin content. This review also insists on the most eco-friendly and innovative extraction methods and their perspective within the next years. The use of bio-based solvents, aqueous two-phase systems and the centrifugal partition chromatography are the most promising processes. The analysis of the global market and multiple applications of fucoxanthin revealed that Asian companies are major actors in the market with macroalgae. In addition, fucoxanthin from microalgae are currently produced in Israel and France, and are mostly authorized in the USA.

## 1. Introduction

Fucoxanthin is the major carotenoid pigment in marine ecosystems, representing 10% of the total carotenoid production [1,2]. This yellow-orange pigment is produced by Chromista algae, a group composed of all brown and golden-brown macro and microalgae such as diatoms, haptophytes, dinophytes and brown seaweeds. Fucoxanthin masks the color of chlorophylls and gives the characteristic brown color to the Chromista. In diatoms, it was proven that fucoxanthin gives the capacity to absorb photons in a wide blue-green spectral range of visible light, which are the most available radiations in the water column [3]. Indeed, there is a lack of red light availability for chlorophylls at certain depths as only blue light can reach further to the cells. Therefore, along with the light-harvesting by chlorophylls, fucoxanthin increases the efficiency of using different light wavelength for carbon fixation.

**Figure 1:**
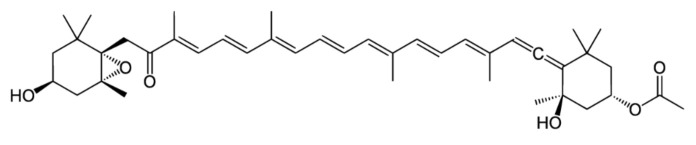
Chemical structure of the fucoxanthin molecule.

Fucoxanthin is part of the carotenoid family named xanthophylls, whose chemical structure is distinct from the second carotenoid family, the carotenes, because they contain oxygen. This particularity along with an epoxide group and a conjugated carbonyl group confer the xanthophylls anti-oxidant properties very demanded in aquaculture, cosmetics and pharmacology [4,5,6]. Fucoxanthin, whose molecular structure was fully described in 1990. [7], is even more valuable in pharmacology as it contains an unusual allenic bond (Figure 1) that is supposed to be the origin of its anti-cancerous, anti-diabetic, anti-obesity, anti-inflammatory, anti-angiogenic, anti-malarial properties and of several protective effects [8,9,10,11,12,13]. These properties are of great interest to the food and health industry, which has increased the global demand for fucoxanthin.

The objectives of this review are multiple. As a first step, a state-of-the-art of the biological function of fucoxanthin in algae was achieved in order to provide a complete overview of this pigment, including its role in the natural environment within the golden-brown macroalgae and microalgae. The latest hypotheses on fucoxanthin synthesis are presented. Then, as a consequence of the growing interest in fucoxanthin in research and industrial fields, especially for its numerous properties (anti-cancer, anti-oxidant, anti-diabetes etc.), this review focuses on several points. The best fucoxanthin producing algae species are listed, the culture parameters of a major influence are described, and the downstream processes are categorized. Due to the high growth, ease of control and optimization in microalgal cultivation, the fucoxanthin production by microalgae has been well documented. The possibility for scaling up both production and productivity processes has already been established. In this review, research developments in production and productivity of fucoxanthin by micro and macroalgae are reported in the last three years. Since the pigments are attached to the chloroplast membrane, the state-of-the-art extraction and purification methods are also presented to find the best methods to extract the high-value substance that can be mass-produced by microalgae, with a focus on eco-friendly downstream processes. Finally, a report of the global market and applications of fucoxanthin in food, cosmetics and medicine is provided.

## 2. Isomers of Fucoxanthin

**Figure 2:**
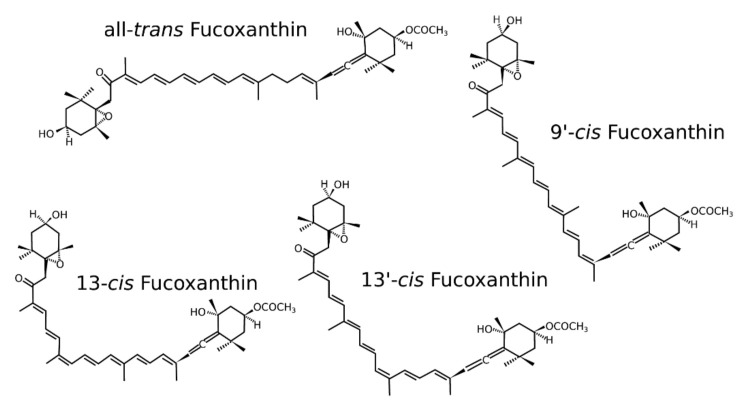
Isomers of fucoxanthin, all-*trans* fucoxanthin, 9′-*cis* fucoxanthin, 13-*cis* fucoxanthin, 13′-*cis* fucoxanthin.

As other carotenoids, fucoxanthin is highly unsaturated, its molecular structure is unstable and very reactive. As a matter of fact, fucoxanthin is likely to form isomers and is sensitive to oxidation, caused by oxygen, other pro-oxidative molecules and sometimes enhanced because of the isomerization [14], to high temperature and to light [15,16,17]. Fucoxanthin possesses four isomers, all-*trans* fucoxanthin, which is the major isomer in the natural environment, 9′-*cis* fucoxanthin, 13-*cis* fucoxanthin and 13′-*cis* fucoxanthin (Figure 2). As it was proven for ß-carotene [18], the *cis* and *trans* isomers of fucoxanthin have different anti-oxidant properties. When increasing the temperature, all-*trans* fucoxanthin changes into the *cis* isomer and losses 21% of its anti-oxidant potential (measured with DDPH test) [19]. This result was counterbalanced with another later study showing that the difference between an antioxidative potential of the four fucoxanthin isomers is not significant [20]. On the contrary, the *cis* forms of fucoxanthin from the seaweed *Undaria pinnatifida* were demonstrated to have better antiproliferative effects against HL-60 cancerous cells, against leukemia cells and Caco-2 (colon cancer) cells [21]. However, the *cis* forms being more sterically hindered, their bioavailability is lower than *trans* forms.

## 3. Biological Function

### 3.1. In Algae

Fucoxanthin is found in light capture apparatus in the photosystems, as part of the electron chain transport on the thylakoid membrane [22]. It is attached to chlorophylls (*a* and *c*) and apoproteins forming complex structures called fucoxanthin-chlorophyll protein complexes (FCP) [23,24]. Due to their particularity of absorbing blue-green spectrum by attaching to chlorophylls, the light-harvesting complexes are capable of a wider light spectrum absorption thanks to fucoxanthin, between 390 and 580 nm [22]. This ability is useful and advantageous for algal cells when there is a lack of red light availability for chlorophylls at certain depths as only blue light can reach further to the cells [23]. Photosynthesis starts with the capture of photons by pigments such as chlorophylls and fucoxanthin, then with the excitation of electrons of the pigments in the photosystem II (PSII) and the subsequent transport of electrons through water splitting at the PSII by various electron carriers in a fixed order. The flow of electrons is passed to plastoquinone, cytochrome b_6_f, plastocyanin and finally to photosystem I (PSI) to produce NADPH that is used in the Calvin-Benson cycle or the carbon fixation cycle [25]. The role of fucoxanthin in this chain transport is to aid chlorophylls capture light photons more efficiently and to protect the oxidative damage induced by light under high intensity [23,26].

Fucoxanthin molecules were classified for the first time in 2009 into three categories with the study of the diatom *Cyclotella meneghiniana*, according to their photon absorption capacities [22]. “Fx-blue” are high energy photons absorbing fucoxanthin molecules strictly absorbing in the blue range of the visible light spectrum (λ_max_ = 463 nm). “Fx-green” are intermediate energy photons absorbing fucoxanthin molecules absorbing in a wider blue/green light range (λ_max_ = 492 nm). Finally, “Fx-red” are low energy photons absorbing fucoxanthin molecules additionally absorbing in an even wider blue/green light range called “red-shifted”, range (λ_max_ = 500–550 nm). This specific shift in absorption properties, which is driven by growing conditions, explained the capacity of fucoxanthin to harvest photons over a wide light spectrum [3]. Though these molecules have the exact same molecular structure (there is only one molecule of fucoxanthin), their energetic level and their absorption capacity are intrinsically linked to their binding site on the FCP as was demonstrated by crystallography in the diatom *Phaeodactylum tricornutum,* which possesses seven fucoxanthin molecules all differentially positioned when bound on the FCP [3]. The overview of several studies concluded that there were, in diatoms, seven fucoxanthin molecules of which two are “Fx-blue”, two are “Fx-green” and three are “Fx-red” [22,27,28,29,30].

Energy transfer from fucoxanthin molecules to Chl *a* was particularly studied [22]. Like peridinin, a carotenoid specific to some dinoflagellates microalgae [31], the most efficient pathway for energy transfer from fucoxanthin starts with the excited state S1/ICT [28]. This state is a coupling of the fucoxanthin singlet (S1) and the intramolecular charge transfer phenomenon (ICT). With their spatial configuration on the FCP, Fx-red are low energy molecules and therefore the most willing to follow the S1/ICT pathway [22]. This gives Fx-red the ability to be particularly efficient in transferring the incident light energy, in comparison with Fx-blue and Fx-green. Thus, Fx-red is able to capture higher light intensities without creating excess undissipated energy which could generate harmful ROS. Fucoxanthin can therefore be considered, in a way, as photoprotective although it is not its main function contrary to diadinoxanthin and diatoxanthin. More precisely, some fucoxanthin molecules are involved in the quenching of the Chl *a* triplet (^3^Chla*) [32]. The quenching is the extinction of fluorescence of the ^3^Chla* in order for Chl *a* to reach its fundamental state, it is the dissipation of the excess energy, generating heat. It is a photoprotection mechanism. Those fucoxanthin molecules are the Fx-red, which efficiently transfer the energy from their singlet state S1/ICT to the ^3^Chla*. Quenching measures of the ^3^Chla* proved that fucoxanthin definitely plays a role in photoprotection. To corroborate this result, another study on *Chaetoceros gracilis* supposed that the light capture represents around 60% of the FCP activities and the quenching around 30%, in low light (LL) conditions [33]. LL condition eliminated the possibility of quenching by the Diadinoxanthin/Diatoxanthin photoprotective cycle, only activated in high light (HL) conditions [34]. Furthermore, no Diatoxanthin was found in cells [33]. This result supported the possibility for another type of quenching, by what was previously considered as only photosynthetic pigments such as fucoxanthin. Finally, a crystallographic study confirmed the presence of several fucoxanthin molecules distinctly positioned (i.e., bound) on the FCP, each one being very close to at least one Chl *a*, substantiating the rapid energy transfer between fucoxanthin and Chl *a* [3]. Complementary to its photosynthetic property, it was proven that independently from the Diadinoxanthin/Diatoxanthin photoprotective cycle, fucoxanthin also plays a role in photoprotection. This role appears as essential even in LL and would be complementary or in support with the Diadinoxanthin/Diatoxanthin cycle which is not continuously activated in thylakoids.

### 3.2. In Human

Many review studies have described the properties of fucoxanthin relative to human health [8,35,36,37,38,39] and cosmetics [9,40,41]. Table 1 presents the latest studies of functional activities of fucoxanthin (2020–2021). In particular, studies about cancer inhibition are part of the major fucoxanthin research concepts with the most occurrences in the literature [42]. However, contrary to many studies that demonstrated the anti-cancer properties of fucoxanthin, Terasaki et al. showed that fucoxanthinol, known to be the real active metabolite of fucoxanthin [43], accelerated the growth of human pancreatic cancer cells by up-regulating the expression of key genes [44]. These results are the opposite of what Terasaki et al. previously showed in mice and hamster pancreatic cancer cells as fucoxanthinol induced apoptosis in cancerous cells [45,46]. Another biological activity of fucoxanthin is anti-inflammatory effects in epithelial cells, macrophages, adipocytes, liver cells, lung cells, fibroblasts, and so forth. A study showed that a methanolic extract of the haptophyte microalgae *Tisochrysis lutea* had a higher anti-inflammatory effect than a pure fucoxanthin extract with the same pigment concentration [47]. Indeed, *T. lutea*—like *Isochrysis galbana,* which is a close strain—possesses, in addition to fucoxanthin, several other phenolic compounds that can have anti-inflammatory properties [48]. On the other side, some studies demonstrated that fucoxanthin can have toxic effects on human cells. Fucoxanthin was shown to reduce PC-12 cells’ (neuronal cells) viability at a concentration higher than 5 μM, to reduce 40% of the viability of human lymphocytes in 24 h at a concentration of 10 μM and to be toxic at a concentration of 40 μM in 16 h for keratinocytes [49,50,51].

## 4. Biosynthesis in Algae

**Figure 3:**
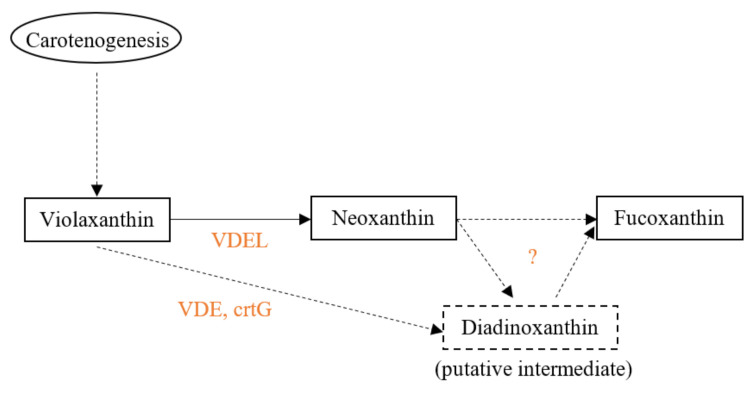
Hypothetic pathway of the fucoxanthin final synthesis reactions. Orange: genes implied in biosynthesis.

The carotenogenesis pathways among algae are not well known; they were proposed according to their similarity to land plants and based on the chemical structure of carotenoids [97]. The precise biosynthesis of fucoxanthin is currently unknown (Figure 3). However, studies have linked Violaxanthine De-Epoxidase (VDE) to the regulation of fucoxanthin production. Kwon et al. showed a correlation between a higher fucoxanthin synthesis and a higher VDE expression, along with a higher expression of the ISPD gene, in the diatom *P. tricornutum* [98]. According to their results, these two genes are critical genes for the regulation of fucoxanthin biosynthesis in *P. tricornutum*. VDE is likely to be part of the upstream regulation of fucoxanthin as it is known to interfere in the de-epoxidation of violaxanthin into antheraxanthin [97]. Furthermore, an enzyme participating in the biosynthesis of fucoxanthin has been characterized by Gaidarenko et al. in the diatom *Thalassiosira pseudonana*, encoded by the Violaxanthine De-Epoxydase Like gene (VDEL) [99]. When the gene was up-regulated, the fucoxanthin content increased and reversely, pigments from the photoprotection cycle such as Diadinoxanthin and Diatoxanthin logically decreased. According to this study, and because the VDEL gene is present in five other diatom species (*Thalassiosira oceanica*, *Cyclotella cryptica*, *Phaeodactylum tricornutum*, *Fragilariopsis cylindrus*, *Pseudo-nitzschia multiseries*), this result is supposed to apply to all diatoms. Based on the assumption of Lohr and Wilhem in 2001, arguing that the violaxanthin was a precursor of Diadinoxanthin, Diatoxanthin and fucoxanthin [100], it is likely that the VDEL enzyme influences not only the violaxanthin content but also the Diadinoxanthin, Diatoxanthin and fucoxanthin content, strengthening the results and the hypothesis of Gaidarenko et al. Furthermore, another research team confirmed the VDEL enzyme is responsible for the central reaction converting the violaxanthin into neoxanthin in Chromista (clade containing diatoms, haptophytes and brown seaweeds) [101]. They also supposed Diadinoxanthin to be an intermediate between neoxanthin and fucoxanthin in contrary with another study assuming that fucoxanthin is synthesized directly from neoxanthin [38], despite the fact that the corresponding enzymes have not been characterized yet. In both cases, the VDEL enzyme could indirectly regulate the production of fucoxanthin (Figure 3). However, in the haptophyte microalgae *T. lutea*, which is one of the most interesting species for fucoxanthin production, no neoxanthin has been found so far [102,103]. It indicates that neoxanthin cannot be considered as a precursor of fucoxanthin in all Chromista species.

A study also highlighted the role of the MET pathway (methylerythritol phosphate pathway) in the accumulation of fucoxanthin in *P. tricornutum* [104]. Indeed, the research team showed that both CMK and CMS genes, involved in the MEP pathway, enhanced the fucoxanthin content in *P. tricornutum* by increasing the photosynthetic efficiency, and by redirecting the metabolic precursors towards fucoxanthin biosynthesis from the lipogenic pathway [104]. Thus, the MET pathway is not directly involved in fucoxanthin biosynthesis but it allows a greater accumulation of this pigment in *P. tricornutum*.

## 5. Bioavailability

Two decades ago, the fate of the degradation products of carotenoids in the human body was unknown [105]. Then, the beneficial effects of some carotenoid metabolites were demonstrated, which motivated further research on this subject. For example, it is the case with fucoxanthin which is mainly ingested through food consumption of macroalgae such as brown seaweeds *Undaria* and *Laminaria* in Japan. Finally, researchers came to understand that the interest of fucoxanthin lays in its bioavailability (the measurement of the rate and speed of absorption by cells) and its metabolization. In mammals, fucoxanthin is first metabolized into fucoxanthinol, then into amarouciaxanthin A [106].

More precisely, once ingested, fucoxanthin is hydrolyzed in fucoxanthinol in the intestinal tract. Then, fucoxanthinol is absorbed by intestinal cells and carried to blood vessels, suggested that the bioavailability of fucoxanthin is indeed based on that of fucoxanthinol in mammals [43]. A study discovered that in some murine liver cells, fucoxanthinol was then metabolized into another molecule, amarouciaxanthin A. This suggestion was then demonstrated on a larger scale by other studies, proving moreover that fucoxanthinol and amarouciaxanthin A were detected in many tissues (plasma, erythrocytes, liver, kidneys, heart, spleen for fucoxanthinol, and adipose tissue for amarouciaxanthin A), unlike fucoxanthin, except for a few traces, simply meaning that a tiny part had not been metabolized [107,108].

## 6. Producing Species

### 6.1. Best Producers

Many algae species are known for their fucoxanthin content in the cell membranes that can be extracted. The current commercial production of fucoxanthin is based on brown seaweeds (macroalgae) [109]. Macroalgae produce fucoxanthin in varying amounts depending on the species and their metabolism. The content may vary from 0,02 to 4,96 mg·g^−1^ in fresh samples of different species (the maximum was reached in *Undaria pinnatifida*) and 0,01–2,08 mg·g^−1^ in dried samples of different species [39]. The concentration of macroalgal fucoxanthin is thus very low and not sufficient for the global demand of this potential nutraceutical compound. Microalgae, on the other hand, show to be a great source of fucoxanthin as they are fast-growth microorganisms and their fucoxanthin content is high. Diatoms and haptophytes are the most promising groups of microalgae for the commercial production of fucoxanthin [109]. For example, the fucoxanthin content can reach as unusually high as 59.2 mg·g^−1^ in dried samples of the diatom *P. tricornutum* [110] and 79.40 mg·g^−1^ in dried samples of the haptophyte *T. lutea* [111] in batch culture under optimized conditions. Diatoms accumulate fucoxanthin in large amounts and have already been widely studied and exploited for fucoxanthin production. Two microalgal diatom species and one haptophyte were identified to produce a very high amount of fucoxanthin, *P. tricornutum*, *Odontella aurita*, and *Isochrysis aff. galbana* (*T. lutea*), respectively [39]. These species have been an extensive target study for fucoxanthin production and optimization for higher productivity in the literature. Table 2 lists some of the species of microalgae that produce fucoxanthin from studies between 2020 and 2022.

At an industrial scale, the main objective is to optimize the production costs constituting the greatest expenses like the labor, energy and chemical supplements [137]. For example, the cost of a culture of *P. tricornutum* required for an equivalent organic weight is four times lower than the other diatom *Chaetoceros gracilis* in hatcheries production [138]. In general, diatoms and haptophytes represent popular species because they are easy to cultivate and can reach a high amount of biomass in a short time. *P. tricornutum* can reach, for instance, a biomass productivity of 1500 mg·L^−1^·day^−1^ in an outdoor helical reactor [139]; *Thalassosira Weissflogii* can reach 538 mg·L^−1^·day^−1^ in a tanks reactor [130]; and *T. lutea* can reach 940 mg·L^−1^·day^−1^ [112] and 350 mg·L^−1^·day^−1^ [114] in 400 mL photobioreactor. Even if most studies are with diatom species in literature, the cultivation of haptophyte species represents a certain advantage in fucoxanthin extraction processes. Indeed, diatoms possess a silica frustule which necessitates an additional step in the extraction process to remove it, whereas non-coccolithophore haptophytes, like *T. lutea* and *Pavlova lutheri*, do not possess one, which eases the extraction of fucoxanthin in the downstream processing [140,141]. *T. lutea* especially is part of the best fucoxanthin producers [39,118]. This species is well known as it is widely used in live feed in aquaculture [142,143] as it possesses valuable nutritional properties attributed to its capacity to accumulate lipids and produce carotenoids [144,145]. Furthermore, *T. lutea* is able to achieve high biomass with high fucoxanthin concentration in a short period of cultivation (between 6 to 15 days). In addition, the culture can be carried out with sustainable and low-risk resources [39,102]. With mild growth culture conditions such as a wide range of light irradiances and temperatures [146,147,148], *T. lutea* is definitely easier to cultivate than other microalgae species.

### 6.2. Measurement of Fucoxanthin

In some studies with a further purpose of the continuous production of microalgae, such as the larvae feeding for example [149], it is common to measure the pigment’s productivity (e.g., mg.L^−1^.day^−1^), also referred to as the production rate or volumetric productivity. The objective is to express the effectiveness of the production of a valuable pigment by combining two main factors being the pigment content in cells and the biomass growth in terms of time and cell density.

However, when the objective is to better understand how to improve the pigment content, by cells, of an algae species, therefore excluding the contribution of the amount of biomass, it is more appropriate to measure the production in mg.g^−1^ or in fg.cell^−1^, also referred as the specific pigment concentration. Indeed, even in a further industrial purpose, the first step is to study the physiology of the algae species as the production of valuable metabolites such as carotenoids often results from stress modifying their metabolism (nutrients, light, temperature, etc.) [104,128,146,150]. Some studies express the pigment production in mg.L^−1^, also referred as the volumetric concentration, which depends on two factors being the pigment content and the biomass in terms of cell density only. This measure is not suitable to describe the physiology of algae cells, as it is not normalized by the cell density. A higher fucoxanthin production in mg.L^−1^ does not necessarily imply that cells produce more fucoxanthin, it might just be the result of an increase of the biomass. However, this measure can be used to compare the production of fucoxanthin in several cultures in batch culturing mode for example.

A lot of studies gather the information by displaying both productivity and production measures. This way to present results allows to consider the influence of the culture conditions simultaneously on the cell metabolism and the relative growth of the studied species (Table 2).

## 7. Culture

### 7.1. Reactors, Temperature, pH and Salinity

**Figure 4:**
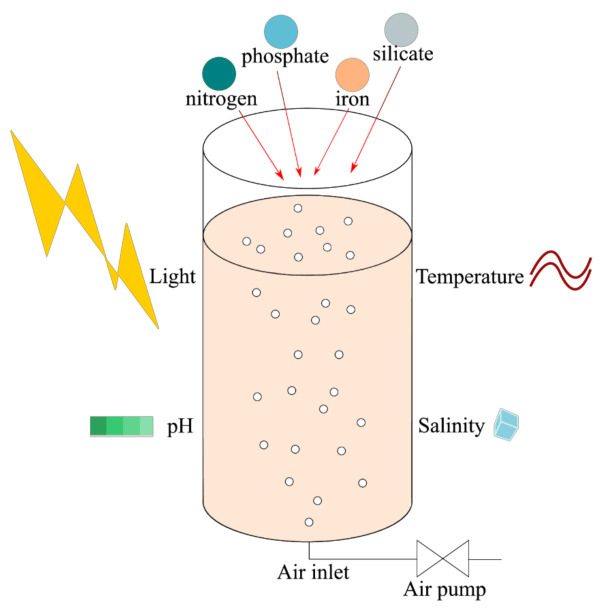
Schematic illustration of a close tubular air-lift photobioreactor and main culture parameters.

Microalgal cultivation has been traditionally operated with open systems such as the open tank and raceway ponds [151]. The open ponds are easy to construct and operate with low cost for mass production of microalgae [152], but also for fucoxanthin production as it has been shown for *P. tricornutum* cultivated in the open pond [153]. However, they pose a risk of limited control of cultures towards the progressive changes of the surrounding environment such as contamination, incremental increase of salinity, the problem of mass transfer and loss of nutrients through evaporation [154]. These drawbacks have shifted interest in microalgae cultivation towards the use of more performant, more controlled close systems, the photobioreactors (PBRs) (Figure 4). The most used PBRs are tubulars, stirred tanks and flat panels. Among them, flat panels are the most preferred in literature [117,155,156,157], and even more to study the specific response of microalgae to light. Indeed, they have a high surface area to volume ratio, and even if the area required for such reactors might be inconvenient at industrial scale, there are advantages. A flat panel reactor permits higher light absorption and reduces the effect of self-shadowing caused by the microalgal growth. Therefore, it allows greater biomass production. On the other hand, as cells produce more fucoxanthin in low light, self-shadowing can also be of interest. Even if it still happens when culture in flat panels reach high concentration, a lack of an early self-shadowing can be inconvenient for fucoxanthin accumulation. The balance between biomass and fucoxanthin accumulation is essential. In addition, the low oxygen accumulation in flat panel reactors prevents the growth of contaminated aerobic microorganisms, reinforced by the easiness of sterilization and the flexible structures [158,159]. A study by Derwenkus et al. analyzed the cost of scaling-up production of both EPA and fucoxanthin in flat-panel airlift PBRs and concluded that the profitability of the process mostly depended on the fucoxanthin market price [122]. For fucoxanthin production, batch cultures are often operated with a short residence time that is less than 15 days [110,121,158,159,160,161]. Fucoxanthin productivity takes into account both the daily production of pigments and the growth of microalgae. It is thus commonly associated with the continuous modes after optimization of the culture parameters [114,121,122,152].

Temperature in the reactor also affects the productivity of fucoxanthin as it is linked to biomass growth. Depending on cultivated species, the optimal temperature can vary in the mesophilic range (30 °C for *T. lutea*, 22 °C for *P. tricornutum*, 23 °C for *Cylindrotheca fusiformis*) [114,160,161]. pH was shown to have an influence on fucoxanthin accumulation in *P. tricornutum* as fucoxanthin is sensitive to low pH media [26]. Wu et al. demonstrated the impact of flocculants, the common microalgae harvesting agents, on the fucoxanthin content of species [162]. The use of a high dose of flocculants increases the recovery rate of biomass but caused a significant reduction of fucoxanthin due to low pH and detrimental effects associated with high valent metallic ions such as aluminum [24]. All of the current most performant species for high fucoxanthin production are marine microalgae whose growth depends on the salinity of the medium which acts as osmotic control of metabolites. The study of Ishika et al. showed that *P. tricornutum* grew well and produced more fucoxanthin at optimal salinity of 45‰, while halophile microalgae accumulated more fucoxanthin at up to 77‰ [152]. Fucoxanthin plays an important role in carbon fixation during photosynthesis and thus has been found to be affected by the mass transfer of carbon [26,110,162]. Agitation and aeration in the reactor could increase the fucoxanthin content and reduce the harvesting time by half as demonstrated in the work of Bauer et al. [26].

### 7.2. Light

One of the most important parameters for fucoxanthin production and productivity is the light condition. Various studies have demonstrated the influence of light wavelength, intensity and the light-darkness cycle on the accumulation of fucoxanthin. Studies have shown that low light intensity (less than 100 mol.m^−2^.s^−1^) promotes the fucoxanthin production [39,114,120,163,164] while high light intensity (starting from 150 mol.m^−2^.s^−1^) can damage the photosystems, therefore activates the production of photo-protective pigments (Diadinoxanthin and Diatoxanthin) [165,166]. Moreover, as more photons are available, cells do not need to capture more photons than necessary due to photon saturation and hence do not produce more chlorophylls and fucoxanthin [167]. Indeed, in the high light regime, not only fucoxanthin degrades [168] but there is also a change of ratio among the photoprotective and the photosynthetic cell pigment content (xanthophylls, carotenoids, chlorophylls). The metabolism behind this is revealed in the violaxanthin and diadinoxanthin cycles that control the production of zeaxanthin and diatoxanthin under high light intensity [169]. In that case, fucoxanthin content is reduced while zeaxanthin, and diatoxanthin, the photoprotective pigments, are accumulated to protect the photosystem [34,163,165,170,171,172].

The increase of fucoxanthin in the low light regime is ascribed to the compensation of low light radiance as fucoxanthin is a part of the light-harvesting antenna that promotes the photon capture for photosynthesis. However, biomass production is found to be inversely proportional to fucoxanthin production as high light promotes the development of the biomass [120]. An improved fucoxanthin productivity results therefore, for each producing species, of a compromise between a high light that allows a great biomass production, and a low light that increases the fucoxanthin production. For example, in cultures of *T. lutea*, the highest fucoxanthin productivity (9.81 mg·L^−1^·day^−1^) was achieved at 300 mol·m^−2^·s^−1^ while the highest fucoxanthin content (5.24 mg·g^−1^) was achieved at 150 mol·m^−2^·s^−1^ [114]. In cultures of the haptophyte *Isochrysis zhangjiangensis*, the highest fucoxanthin productivity (3.06 mg·L^−1^·day^−1^) was achieved at 100 mol·m^−2^·s^−1^ while the highest production (22.6 mg·g^−1^) was achieved at 40 mol·m^−2^·s^−1^ [120].

Fucoxanthin absorbs blue-green light and thus has shown, in diatoms, to be accumulated in blue light even at high intensity while red light favor the production of other pigments such as diadinoxanthin [121,161]. Furthermore, in the diatom *Cylindrotheca closterium*, red light and green light decreased the fucoxanthin content because of the lack of blue light [173]. In *T. lutea*, the highest fucoxanthin content was found in blue-green light while the highest fucoxanthin productivity was found in red-blue-green light (which is closer to white light) [116]. The accumulation of fucoxanthin in blue-green light was expected, as this spectrum is prevalent in the natural habitat of brown algae [3]. Indeed, the absorbance spectrum of fucoxanthin ranges from 450 to 540 nm in solution [174]. This range is even wider, between 390 and 580 nm, once the fucoxanthin is bound to a FCP [22].

### 7.3. Nutrients

Nutrients play an important role in microalgae growth as they are required to synthesize lipids and macromolecules such as nucleic acids and proteins that are essential for algal cells [175]. Nitrate, phosphate, iron, and silicate (for diatoms) have been identified in various studies as nutrients that have an impact on cells growth and on biomass accumulation [26,135,158,161,162,176]. 

#### 7.3.1. Nitrogen

Nitrogen is vital to microalgae as it is incorporated into macromolecules such as proteins, enzymes and nucleic acids, which have a structural or functioning role in cells. Nitrogen can be supplied from an inorganic source either as ammonium or nitrate [135,176,177]. Nitrate is the main form of oxidized nitrogen which accumulates in algae [178]. Subsequently, nitrate is converted into ammonium which is easier to be assimilated into large molecules through transamination in the anabolic pathways [179]. Nevertheless, ammonium when used as a feeding source decreases the pH medium [180], which can hamper the growth of microalgae due to pH-sensitive proteins and enzymes. Many studies have demonstrated the strong correlation between nitrate concentration and biomass production, which indicates nitrogen is one of the limiting factors for biomass production [181,182,183,184,185,186]. High nitrate content promotes the growth of microalgae while a low concentration of nitrate inhibits the growth. Nitrate also has an influence on the pigment content as porphyrins, the core of chlorophylls, contain nitrogen [26,158]. The study investigated by Gao et al. showed the positive linear correlation between chlorophyll and fucoxanthin in *T. lutea*, which explains the indirect dependence of fucoxanthin on the nitrate concentration in the medium [115]. Studies have shown high dependence of fucoxanthin on nitrate concentration in various microalgae species. However, in batch culturing mode, a higher nitrate concentration can lead to a higher biomass concentration which induces the self-shading of the cells. With less access to incident light, the photosynthetic pigments such as fucoxanthin are increased. Therefore, in some studies with batch culturing mode, higher fucoxanthin concentration is linked to higher biomass and not directly to a higher nitrate concentration [110,184,185,186,187]. In Xia et al. however, two batch mode cultures with 6mM and 18mM of nitrogen respectively resulted in the same biomass concentration after 12 days of culture in low light, but the 18mM nitrogen culture resulted in the highest fucoxanthin content [155]. In this case, we can effectively attribute the increase in fucoxanthin content to a higher nitrogen concentration. As to fucoxanthin production, in mg·L^−1^, or fucoxanthin productivity, in mg·L^−1^·day^−1^, it is effectively promoted at high nitrate level due to the increase of the biomass, while too low nitrate causes a decline [110,111,160]. Xia et al. investigated the effect of the initial concentration of nitrate as a feeding source on fucoxanthin content [188]. The initial high concentration of nitrate boosts the accumulation of fucoxanthin in the chloroplast membranes compared to the initial low nitrate. Furthermore, initial high nitrate coupled with nitrogen supplement increases fucoxanthin content more than four-fold as much as only using initial low nitrate [141]. Supplement of nitrogen has been demonstrated to augment two-fold or up to three-fold fucoxanthin content in *P. tricornutum* [110]. On the contrary, nitrogen deficiency induces the degradation of compounds containing nitrogen like proteins and chlorophylls, and therefore decreases the photosynthetic capacities [189].

Further investigation showed the medium type also influences the fucoxanthin accumulation in microalgae [26,190]. Conway medium was shown to be more effective than f/2 medium in inducing fucoxanthin production in the work of Gómez-Loredo et al. [176]. The high performance of Conway medium is due to a higher concentration of nitrate which is sufficient for microalgae to keep high content of fucoxanthin during the whole cultivation period [110,176]. Nitrate influence on fucoxanthin has been found further to be a synergic effect with light and salinity [188]. Combination of both light availability and nitrate level can increase further fucoxanthin quantity in *Cyclotella cryptica* and an increase of both the nitrate level and the salinity at optimal conditions enhances fucoxanthin content in *T. lutea* [111]. The synergic effect of nitrate level on fucoxanthin could be exploited further for high production of fucoxanthin.

#### 7.3.2. Phosphorus

Phosphorus is essential for microalgae as it is a part of the backbone of nucleic acids and ATP, the energy carrier of cells, and phospholipids [175]. Contrary to nitrogen, phosphorus does not have a high influence on biomass growth and fucoxanthin accumulation as shown in the work of Lu et al. and Sun et al. [141,161]. Very few studies have demonstrated the correlation between phosphorus and fucoxanthin content in contrast to the well-documented nitrogen influence [141,161]. 

#### 7.3.3. Silicate

In diatoms, another important nutrient is silicate which is required for cell growth as the cell wall contains silicon. Therefore, silicate availability is linked to the morphology of the diatom cells which can have impacts on cell growth and eventually fucoxanthin content [133]. High silicate level promotes the growth of both biomass and fucoxanthin accumulation in diatoms [133,134,191]. The enhancement of fucoxanthin is due to the upregulation of metabolism and cell cycle progression associated with silicate in marine diatoms [133]. The study of Mao et al. showed that at a high level, silicate induces the accumulation of fucoxanthin but at a low level it suppresses the fucoxanthin production [133].

#### 7.3.4. Carbon

Mixotrophy is considered the mixed condition between photoautotrophy and heterotrophy. In mixotrophy, microalgae can use either light or organic carbon as energy sources and carbon sources can be supplied through carbon dioxide sequestration or from organic sources [192,193]. The ease of shifting towards different sources makes the algae more independent of light, which does not limit the culture growth as in autotrophy and assimilation of carbon can be supported by organic substances similar to heterotrophy. By taking advantage of both photoautotrophic and heterotrophic modes, mixotrophy utilizes the resources more efficiently and for high biomass production. As for nitrogen, a higher biomass may induce the self-shading of cells and subsequently may enhance the production of photosynthetic pigments such as fucoxanthin. Alkhamis et al. reported a two and a half times increase of fucoxanthin content in the mixotrophic culture of *T. lutea* (11.5 mg·g^−1^ DW) compared with the phototrophic culture (4.8 mg·g^−1^ DW) [146]. In a mixotrophic culture of the diatom *Nitzschia laevis*, the fucoxanthin content reached 15.6 mg·g^−1^ DW while in heterotrophic culture it reached 10 mg·g^−1^ DW [194]. Mixotrophic cultures of *P. tricornutum* have been investigated on the high accumulation of fucoxanthin in microalgae and have shown promising results for both biomass and fucoxanthin production, which can be optimized for optimal production of the xanthophyll pigment [121]. These results prove that the mixotrophic cultivation strategies for some microalgae could be more suitable for both biomass and fucoxanthin accumulation. At an industrial scale, one limit of the mixotrophy culture could be the cost of the organic carbon source that is used (glycerol, starch, spruce hydrolysates) [195,196,197].

## 8. Extraction and Purification

### 8.1. Ultrasound Pretreatment

Because fucoxanthin is attached to proteins and other pigments such as chlorophylls in the light-harvesting system anchored inside the membrane, its extraction is improved when pretreatment of algal biomass is conducted prior to or during extraction [198,199,200]. Especially, diatoms possess a thick cell wall made of silica that needs to be disrupted to achieve an efficient fucoxanthin extraction. The degradation of cell walls and membranes can be achieved either by mechanical, chemical, thermochemical, biological or electromagnetic methods [201]. In contrast, the haptophyte microalgae *T. lutea* does not possess a silica cell wall. Therefore, this species is not only one of the best producers but is also advantageous for fucoxanthin extraction as it does not require a specific pretreatment. Ultrasound (US) treatment can however be implemented to enhance the extraction step [103]. 

### 8.2. Conventional Solvents

Fucoxanthin is often extracted with conventional methods using organic solvents or water such as maceration [198,202]. As fucoxanthin contains oxygen and other polar functional groups, its extraction is conducted with medium polarity solvents such as ethanol, acetone, and so forth. Although water has a high dielectric constant, fucoxanthin is barely extracted with hydrotreatment [202,203]. Guler et al. showed that a mixture of tetrahydrofuran and dichloromethane is the best solvent system for fucoxanthin separation from the membrane, but these solvents have a high risk of flammability and explosion (formation of peroxide in presence of air or tetrahydrofuran) [203]. However, two alcohols are often preferred for fucoxanthin extraction. These are methanol and ethanol, which are less toxic compared with tetrahydrofuran and dichloromethane. Indeed, they are safer to use and have shown to have superior performance over carbonyls compounds such as acetone (only one-third yield compared to ethanol) and ethyl acetate (one-sixth compared to ethanol) [13,155,199,202,203]. Due to toxicity and environmental issues linked to methanol, ethanol is preferred and has been demonstrated to extract fucoxanthin efficiently at moderate temperature [13,202,204]. Although the conventional solvents were proven to efficiently extract and recover the carotenoid, they are often time-consuming and require a substantial amount of solvents to be able to remove fucoxanthin from the light-harvesting system attached to the membrane.

### 8.3. Microwave, US, Pressurized Liquid, Enzyme, Sub and Supercritical Fluid, Electrotechnology

Accelerated extraction methods can be listed as follows: microwave-assisted extraction [201,205,206], ultrasound-assisted extraction [198,205], pressurized liquid extraction [198,207,208], high pressure homogenization [209,210] and enzymatic-assisted extraction [211]. They provide rapid and efficient extraction which can preserve pigments without structure modification due to prolonged contact at high temperature. Subcritical and supercritical fluids have also been considered as they have high recovery in fucoxanthin and are more environment-friendly due to less consumed amount of organic solvents, easy to be removed and the use of non-toxic supercritical carbon dioxide but they present lower selectivity among the pigments and higher operation cost as main drawbacks [199,202,203,212]. Electrotechnologies-assisted methods, such as pulsed electric field [213,214], moderate electric field [215], high-voltage electric discharges [216] and electropermeabilization-assisted liquid biphasic flotation [217], provide a good extraction of intracellular components by delivering electric current and allowing an electroporation which increases solvent permeability and hence increases pigment extraction efficiency [218]. However, no studies have been conducted to extract fucoxanthin neither from macroalgae nor microalgae using these advanced methods [202]. 

### 8.4. Bio-Based Solvents

#### 8.4.1. Edible Oil

Due to the use of petrochemical and toxic organic solvents for extraction, bio-based solvents have been proposed such as edible oils, ionic liquids and natural deep eutectic mixtures [199]. Edible oils have the advantage to be already accepted by the consumer, being non-toxic, bio-based and eco-friendly [199]. To our knowledge, only one study reported the effective extraction of fucoxanthin with edible oil from *Sargassum horneri* [219]. The best extraction rate was achieved with short and medium-chain triacylglycerols (SCT and MCT). Indeed, as the main inconvenient of using edible oil is their high viscosity due to carbon chain, thus preventing the diffusion of carotenoids into the oil, it is better to select oils with a minimum number of carbons such as SCT and MCT (from C3 to C8) [219]. Furthermore, it was demonstrated that the absorption rate and thus the bioavailability of fucoxanthin was enhanced when the ingestion was supplemented with MCT from fish oil [220]. 

#### 8.4.2. Ionic Liquids

Ionic liquids are also considered for a more eco-friendly extraction of fucoxanthin. Ionic liquids are solutions of salt with a melting point close to or under ambient temperature, they release anions and/or cations that interact with solutes. So far, the imidazolium-based, the ammonium-based and the phosphonium-based ionic liquids are the most current, though this extraction technique is emerging [221]. In addition to the fact that these solvents are effective at a relatively low temperature, they are non-volatiles. However, they are not bio-based and their cost price is high, hence the need to recycle them by crystallization for example. Table 3 presents some carotenoids extractions using ionic liquids.

#### 8.4.3. Natural Deep Eutectic Solvents

Finally, natural deep eutectic solvents (NADES) are another promising alternative to volatile organic solvents. They are considered as eco-friendly because they are non-toxic, biodegradable and recyclable [223,224]. NADES are composed of two or more natural pure compounds which, together, behave like a unique and pure compound. They have been used for the extraction of astaxanthin, a carotenoid [225]. To our knowledge, in the available literature, there exists only one study suggesting the use of NADES for fucoxanthin extraction from brown algae—*Fucus vesiculosus* [226], combined with ultrasound-assisted extraction (although this step is necessary for macroalgae, it might not be for a non-calcifying microalgae). The research team used one NADES composed of lactic acid and choline chloride (3:1 molar rate), and another one composed of lactic acid, glucose and H_2_O (5:1:3 molar rate). They further analyze the anti-oxidant activity of fucoxanthin, which was not altered by the extraction process. Their results highlighted a great potential for the extraction of fucoxanthin.

In conclusion, conventional solvents such as acetone and ethanol are currently the most used for fucoxanthin extraction, with a preference for ethanol. However, for a few years, more eco-friendly extraction methods are tested for a high recovery of fucoxanthin, one of the most promising being the NADES extraction.

### 8.5. HPLC and Supercritical Anti-Solvent

Gallego et al. (2020) investigated the simultaneous pressurized liquid extraction and purification with adsorbents during extraction of fucoxanthin from *T. lutea* [227]. Results from high-performance liquid chromatography (HPLC) and mass spectroscopy showed that chlorophylls and other xanthophylls are effectively retained by adsorbents and removed from pigment extracts which left only fucoxanthin as the main component although no purity was specified [227]. Chen et al. considered a green process using supercritical anti-solvent fucoxanthin crystallization from *H. mitchellae* using supercritical carbon dioxide with a high recovery and purity of fucoxanthin of 98.3% and 87.7%, respectively [228]. Despite the benefits and advantages of these new methods that utilize less toxic solvent and are less time-consuming compared to traditional procedures, the chromatography systems still remain the most efficient method for purification following extraction.

### 8.6. Aqueous Two-Phase System

Other promising and potential methods are alcohol-salt aqueous two-phases systems (ATPS) or coupling ATPS with ultrafiltration [229,230]. Indeed, these methods are considered eco-friendly as they imply the use of aqueous non-polluting solvents. ATPS is based on the difference of partition coefficient of fucoxanthin between the two immiscible solvents, water being the main component of both phases [231,232,233]. There exist different types of ATPS, the most common being the polymer-polymer system (polyethylene glycol, dextran) [234,235] and the polymer-salt system (phosphate, sulfate, citrate) [236,237]. Other types include the alcohol-salt systems (ethanol, propanol) [238], the micellar/reverse micellar system, the ionic liquids system (imidazolium). In particular, Gómez-Loredo et al. managed to purify fucoxanthin with an alcohol-salt system made of ethanol-K_2_HPO_4_ [239]. They obtained 95.36% of recovery and 66.01% purity for fucoxanthin extracted from *P. tricornutum* and 89.18% recovery and 78.14% purity for fucoxanthin extracted from *I. galbana* [239].

### 8.7. Centrifugal Partition Chromatography

**Figure 5:**
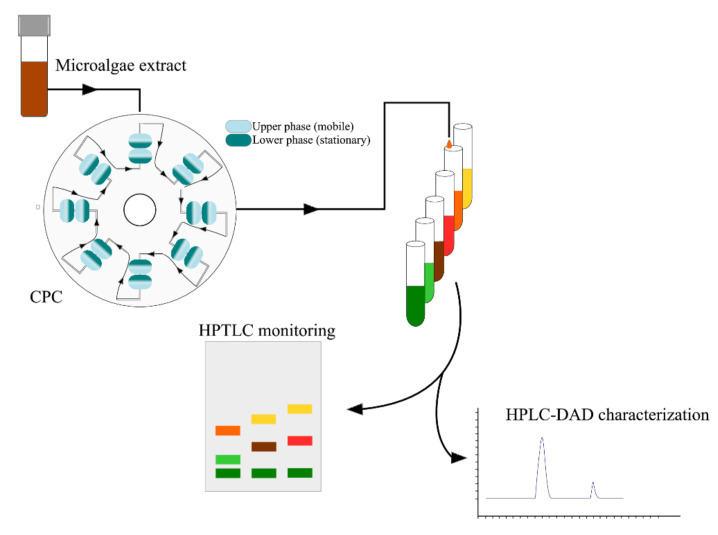
Example of a centrifugal partition chromatography purification process for the pigment separation of an extract of *T. lutea*.

One of the advanced methods has made the purity of fucoxanthin higher than 99% feasible with centrifugal partition chromatography (CPC), after extraction of fucoxanthin with conventional solvent or other methods [103]. The principle of CPC is based on the difference of partition of solutes between two immiscible liquids, which can enrich fucoxanthin and remove most of the impurities [103]. The advantages attributed to CPC compared to silica gel column are that it does not generate polluted silica particles, does not require a high amount of solvents and provides a fast and complete sample recovering without degradation [103]. This technique has made highly purified fucoxanthin feasible and attainable for its eventual applications in food and drugs, and has removed the purification limit of fucoxanthin that was previously only attainable at 99% with tedious procedures [103]. Figure 5 illustrates a purification process of fucoxanthin using CPC method for *T. lutea* culture. There are only two reports for the purification of fucoxanthin so far in the literature to our knowledge [103,240]. According to the study of Gonçalves de Oliveira Junior et al., their first results with *T. lutea* validate the use of CPC as an eco-friendly method for very efficient extraction and pre-purification of fucoxanthin from microalgae. Furthermore, they are convinced that the separation conditions can be optimized.

In addition, all the fucoxanthin extraction methods do not lead to the same purity. Some methods require long and stepwise treatment that increase the impurity content while others are operated at a prolonged high temperature which may cause fucoxanthin degradation. Other influencing factors that induce fucoxanthin decomposition include low pH solution, exposure to light which have to be considered when the purification step is conducted. 

To be able to be used in pharmaceuticals and nutraceuticals, high purity of fucoxanthin is demanded. Oral studies for fucoxanthin activities and toxicity employ a minimum content of 95% fucoxanthin to reduce side effects of impurities [241,242]. Toxicological studies showed that there are known negative effects of fucoxanthin at high concentrations on human cells’ viability such as neuronal cells (PC-12 cells) and keratinocytes (HaCaT). Thus, fucoxanthin purity and concentration need to be monitored [49]. Only the work of Alghazwi et al. specified the purity of fucoxanthin, which is 95%, and its limit concentration to cause toxicity [49].

### 8.8. Stability of Fucoxanthin

Due to the extended conjugated system of double bonds, fucoxanthin is susceptible to isomerism during processing [20,243]. Furthermore, large scale production, stability and long-term storage of fucoxanthin is of importance. Fucoxanthin has been reported to be quite unstable after its removal from the biomass under external conditions such as light, presence of oxygen, high temperature, low pH media and water presence [13,19,26,244]. Fucoxanthin has been known to be easily damaged in water at 26 °C with 30% loss after four weeks [244]. Due to these reasons, fucoxanthin degradation is possible and can be significant in extraction and purification steps. New methods that operate at lower temperatures, lower pressures and avoid long residence time are thus required. A further alternative could be to integrate an anaerobic method to avoid oxidation damage. Another problem of fucoxanthin in applications in pharmaceuticals and nutraceuticals is that fucoxanthin has low bioaccessibility due to low solubility in water and due to its degradation into fucoxanthinol in the case of direct consumption [244]. Encapsulation of fucoxanthin has been well studied to conserve the structure of the xanthophyll and increases its stability as well as its bioavailability [209,210]. Edible materials with high performance such as fatty acids, porous starch, casein, chitosan, gold, have been used to encapsulate fucoxanthin in the form of micro or nanoparticles and showed the improved activity and stability as well as bioaccessibility of fucoxanthin [162,244,245,246,247,248,249,250,251,252,253]. The balance between a preserved stability and an improved activity of fucoxanthin induces a higher cost of the production process due to the complexity of the encapsulation.

## 9. Global Market of Fucoxanthin

**Figure 6:**
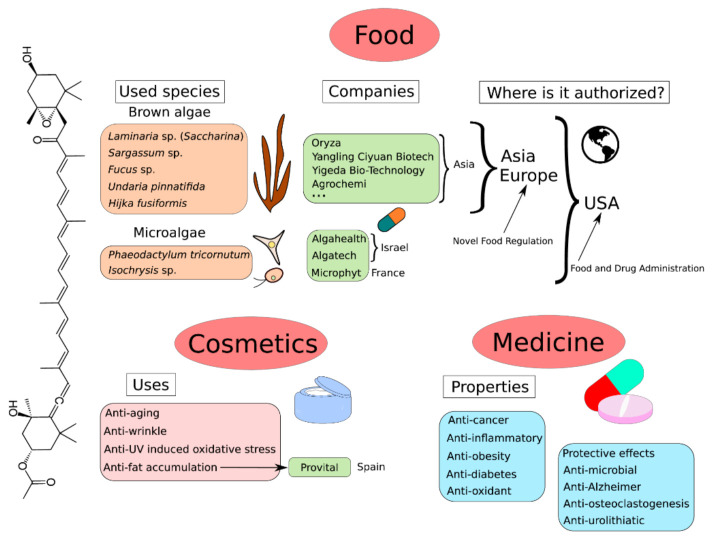
Applications and current market of fucoxanthin in food, cosmetics and medicine.

The multiple studies on fucoxanthin demonstrated that this pigment is an eco-friendly alternative, as a natural ingredient, to food complements, cosmetic compounds and medicines (Figure 6). Because of its unusual chemical structure, the chemical synthesis of the fucoxanthin molecule is not yet possible. Currently, the commercial sources of fucoxanthin for food are brown seaweeds such as *Laminaria* sp. (known as *Saccharina* sp.), *Sargassum* sp., *Fucus* sp., *Undaria pinnatifida* and *Hijka fusiformis* [254]. Fucoxanthin is currently mostly sold in Asia (Figure 6) as brown seaweed powder or oil mostly in capsule or cachet, presented as a fucoxanthin concentrate or in combination with poly-unsaturated fatty acids which are high-value components too. An important number of Asian companies are already major actors in the market (Oryza Oil and Fat Chemical Co., Yangling Ciyuan Biotech Co., Yigeda Bio-Technology Co., Agrochemi Co., etc.). The Novel Food Regulation in Europe authorizes the consumption of the macroalgae species previously described, whereas it authorizes only one fucoxanthin-producing microalgae so far, the diatom *O. aurita* [255]. However, *O. aurita* is currently sold as an organic silicium complement, not as a fucoxanthin concentrate, and only two companies are reported to propose such products to our knowledge (Algosud and Motima Laboratory). In the United States, more products derived from microalgae have been authorized for the market (Figure 6): Fucovital^TM^ by Algatech, NutriXanthin^TM^ and DermaXanthin^TM^ by Algahealth, BrainPhyt^TM^ and Phaeosol^TM^ by Microphyt (which obtained in 2019 the status of New Dietary Ingredient from the American Food and Drug Administration). Furthermore, although some microalgae are great fucoxanthin producers (diatoms and haptophytes), no extraction method has been standardized yet [40]. It represents a major challenge for fucoxanthin to be considered as an economic natural ingredient, as it should be obtained by using simple, fast, and low-cost technologies methods [40]. However, the current unrestricted consumption of fucoxanthin as a dietary supplement, easily accessible online, is an issue. Indeed, a significant number of products are mislabeled/misbranded, highlighting the need for a more stringent regulation [256].

Fucoxanthin represents an important market as a nutraceutical for its numerous health properties (anti-oxidant, anti-cancer, anti-diabetes, etc.) [257], in medicine, and also in cosmetics (Figure 6). Indeed, although it is only a secondary market for now, the bioactivities of fucoxanthin demonstrated so far for the cosmetic industry are anti-aging, anti-wrinkle, anti-UV-induced oxidative stress [9,258,259]. These properties are mainly due to the anti-oxidant activity of the molecule, which prevents from the formation of reactive-oxygenic species (ROS). Indeed, oxidative stress plays an important role in the wrinkle formation process by causing the degradation of collagen matrix and therefore wrinkles [260]. Furthermore, it is known that the UCP1 protein (uncoupling protein 1) prevents the accumulation of adipocytes specifically in brown adipose tissue. In vivo studies on mouse models suggest that fucoxanthin allows the expression of UCP1 in white adipose tissue [261,262], which is the major fatty tissue of adult humans, especially in obesity cases where the brown adipose tissue is atrophied [263]. The UCP1 would then prevent the accumulation of fat, reducing the fat mass of white adipose tissue. Provital, a Spanish company, performed a study demonstrating that after the application of fucoxanthin enriched cream-gel on fatty tissues in 61 volunteers, the fat layer and cellulite were reduced [264]. In response to the increased interest of this carotenoid, the annual growth rate of the current market of fucoxanthin as a nutraceutical or cosmetic ingredient is +2.47% in average. The market size of fucoxanthin was 92 million $ in 2014, 99 million $ in 2017, and is expected to reach US$ 120 million by 2022 [40,265]. Derwenkus et al. concluded that the profitability of the process to obtain fucoxanthin mostly depended on the fucoxanthin market price [122]. They also highlighted the fact that the biomass production cost of microalgae is lower in higher culture volume (from 882 €/kg to 228 €/kg from 0.7 t/year to 170 t/year). 

## 10. Perspectives and Conclusions

Along with other photosynthetic and photoprotective pigments, fucoxanthin is bound to the Fucoxanthin Chlorophyll *a/c* binding Protein and forms the light-harvesting antenna in the chloroplasts of brown macroalgae and microalgae. In addition to its main role of capturing photons, fucoxanthin is a strong anti-oxidant in algal cells by limiting the formation of reactive oxygen species. This property is also exploited in human health, as well as anti-cancer, anti-inflammatory, anti-diabetic and anti-obesity properties. As fucoxanthin cannot currently be artificially synthesized, its production lays on brown algae. The best producers are microalgae, and especially *Tisochrysis lutea*, *Phaeodactylum tricornutum*, *Odontella aurita*. These species are relatively easy to grow as they do not require very specific culture conditions apart from main nutrients (nitrogen, phosphorus, silicate). The main challenge for fucoxanthin production is the design of the photobioreactor, which has to meet several requirements. Cells need to have enough access to light to grow but the irradiance must be low enough to avoid photoinhibition and encourage fucoxanthin accumulation. Furthermore, the reactor must be sufficiently aerated to provide the best balance between oxygen and CO_2_ and adjust the culture pH to its optimum. Currently, the most popular reactor in the literature is the flat-panel photobioreactor which provides homogeneous access to light, but whose main issue is to require a great area for a small culture volume. It can be an important inconvenient at industrial scale. 

The downstream processes to extract fucoxanthin from the culture became a real challenge as they must respect a maximum of green chemistry principles to last in the future. Over the last years, several techniques were tested such as the microwave extraction, the pressurized liquid extraction, the sub and supercritical fluid extraction. These are promising techniques, and more studies will be performed in the years to come, to confirm the results obtained and improve the pigment selectivity. Conventional solvent extractions with tetrahydrofuran, dichloromethane or methanol, are the most described in literature. Nowadays, these solvents tend to be replaced by ethanol which is a more eco-friendly solvent and which showed promising results with a good pigment selectivity and a high extraction yield. Bio-based solvents such as edible oil, ionic liquids and natural deep eutectic solvents are also greatly considered as an ecological alternative for fucoxanthin extraction. The main challenge today is therefore to find the good balance between a high pigment selectivity for fucoxanthin, a high extraction yield, and a process that tends to fully respect the green chemistry principles. Following fucoxanthin extraction, purification processes can be performed. The most promising method seems to be the centrifugal partition chromatography, as this technique relies on a solvent system that can resemble to green solvents used for extraction. Furthermore, it requires a small amount of solvents, is fast, and has already resulted in fucoxanthin fractions purified at >99%.

Today, fucoxanthin is an emerging market specialized in pharmaceutical/nutraceutical with an increasing interest in the cosmetic industry. To our knowledge, no studies have demonstrated an interest in fucoxanthin for animal feeding (other than in aquaculture with the direct consumption of microalgae), nor as a coloring agent in a food or non-food. So far, there are only a few companies commercializing fucoxanthin. For both the pharmaceutical/nutraceutical and cosmetic markets, the products appear to be either a biomass extract of algae, or a purified fucoxanthin extract. In both cases, some products are also valued with the association of poly-unsaturated fatty acids.

## Figures and Tables

**Table 1:** Biological activities of fucoxanthin—studies of 2020–2021. The notation “p“ means the fucoxanthin was purchased and not directly purified from algae within the study.

Property	Source of Fucoxanthin	Target	Reference
Anti-cancer	p	Mice pancreatic cancer cells	[45]
	p	Hamster pancreatic cancer cells	[46]
	p	Mice colorectal cancer cells	[52,53]
	p	Human colon cancer cells	[54,55]
	p	Oral squamous cancer cells (KB)	[56]
	p	Human glioblastoma cells (U87MG)	[57]
	*Laminaria japonica*	Human lung cancer cells	[58]
	p	Human lung and cervical cancer cells	[59]
	p	Nasopharyngeal carcinoma cells	[60]
Anti-inflammatory	p	Inflammation of mice tracheal epithelial cells	[61]
	p	Inflammation in non-alcoholic fatty liver disease	[62,63]
	*Sargassum fusiformis*	Particulate matter-induced inflammation	[64]
	*Tisochrysis lutea*, brown seaweeds	Lipopolysaccharide (LPS)- stimulated RAW264.7 macrophages	[47,65,66]
	p	Neuroinflammatory response in induced-Parkinson’s disease	[67]
	p	Acute lung injury inflammation	[68]
	*Cylindrotheca closterium*	Immunocytes, enterocytes, mesenchymal stem cells	[69]
	*Phaeodactylum tricornutum*	Pro-inflammatory cytokines	[70]
	p	PAMP lipopolysaccharide-induced uveitis inflammation	[71]
Anti-obesity	p	Insulin resistance of obese mice	[72]
	*Sargassum siliquosum*	Diet-induced obesity in rats	[73]
	p	Gut microbiota in high-fat diet-fed mice	[74]
	*Plocamium telfairiae*	High-fat diet-fed mice	[75]
Anti-diabetes	*Sargassum angustifolium*	Streptozotocin-nicotinamide-induced type 2 diabetic mice	[76]
Protective effects	p	dexamethasone-induced myotubes atrophy	[77]
	p	Neurodegenerative disorders	[78]
	p	6-OHDA-Induced Neurotoxicity	[79]
	*Sargassum honeri*	Methamphetamine-induced neurotoxicity	[80]
	p	High glucose-induced oxidative stress	[81]
	p	Irradiated mice	[82]
	p	Calcification of heart valve interstitial cells	[83]
	p	Macular degeneration and retinal pigment epithelial cell senescence	[84]
	p	Atopic dermatitis symptoms	[85]
	p	Fibroblasts cellular senescence	[86]
	p	Ischemia-reperfusion injury in kidney	[87]
	p	UV-B irradiation induced retinal Müller cells	[88]
Anti-oxidant		20-year meta-analysis review	[89]
Anti-microbial	*Thalassiosira* sp., *Chaetoceroes* sp.	Pathogenic bacteria (*Staphylococcus aureus*, *Escherichia coli)*	[90]
	p	20 bacterial species (*Streptococcus agalactiae*, *Staphylococcus epidermidis*…)	[91]
	p	review	[92]
Anti-Alzheimer	*Sargassum horneri*	A*β* oligomers-induced neurotoxicity	[93]
Anti-osteoclastogenesis	p	MAP kinase, Nrf2 signaling	[94]
Anti-urolithiatic	p	Ethylene glycol-induced renal calculus in rats	[95]
Anti-fibrogenic	p	Hepatic stellate cells	[96]

**Table 2:** Microalgae species producing fucoxanthin in dried and fresh sample condition—studies of 2020–2022. The notation “-“ means that the data was not available.

Species		Fx Content (mg·g^−1^ Dry Weight)	Fx Productivity (mg·L^−1^·Day^−1^)	Condition	Reference
*Tisochrysis lutea*	haptophyte	16.05	13.75	dried	[112]
*Tisochrysis lutea*	haptophyte	6.66	1.82	dried	[113]
*Tisochrysis lutea*	haptophyte	10.01	9.81	dried	[114]
*Tisochrysis lutea*	haptophyte	13.09	-	dried	[115]
*Tisochrysis lutea*	haptophyte	16.30	2.77	dried	[116]
*Tisochrysis lutea*	haptophyte	17.80	1.14	dried	[117]
*Tisochrysis lutea*	haptophyte	79.40	-	dried	[111]
*Tisochrysis lutea*	haptophyte	5.40	-	dried	[118]
*Pavlova lutheri*	haptophyte	20.86	4.88	dried	[119]
*Isochrysis zhangjiangensis*	haptophyte	22.6	3.06	dried	[120]
*Phaeodactylum tricornutum*	diatom	13.30	1.41	dried	[117]
*Phaeodactylum tricornutum*	diatom	7.00	-	dried	[118]
*Phaeodactylum tricornutum*	diatom	13.00	8.22	dried	[121]
*Phaeodactylum tricornutum*	diatom	16.30	-	dried	[122]
*Phaeodactylum tricornutum*	diatom	17.55	-	dried	[123]
*Phaeodactylum tricornutum*	diatom	16.13	-	dried	[124]
*Phaeodactylum tricornutum*	diatom	21.90	-	fresh	[125]
*Phaeodactylum tricornutum*	diatom	21.20	-	dried	[126]
*Chaetoceros calcitrans*	diatom	17.51	-	dried	[127]
*Stauroneis sp.*	diatom	11.80	-	dried	[128]
*Stauroneis sp.*	diatom	5.90	-	dried	[129]
*Thalassiosira weissflogii*	diatom	9.00	5.10	dried	[130]
*Odontella aurita*	diatom	16.20	9.41	dried	[131]
*Amphora capitellata*	diatom	41.83	-	dried	[132]
*Nitzschia laevis*	diatom	12.20	-	dried	[133]
*Conticribra weissflogii*	diatom	10.00	-	fresh	[134]
*Sellaphora minima*	diatom	7.60	1.2	fresh	[135]
*Nitzschia paela*	diatom	5.70	0.60	fresh	[135]
*Chaetoceros gracilis*	diatom	15.4	3.82	fresh	[136]

**Table 3:** Carotenoids extraction with ionic liquids.

Salt	Carotenoids Extracted	Reference
DACARB (diallyammonium diallycarbamate)	Fucoxanthin	[127]
BF_4_^−^ (tetrafluoborate)	Astaxanthin	[222]
MS^−^ (methylsulfate)	Astaxanthin	[222]
ethanol + 1-n-butyl-3-methylimidazolium + Br^−^	Astaxanthin	[221]
